# How to use pen and paper tasks to aid tremor diagnosis in the clinic

**DOI:** 10.1136/practneurol-2017-001719

**Published:** 2017-08-26

**Authors:** Jane Alty, Jeremy Cosgrove, Deborah Thorpe, Peter Kempster

**Affiliations:** 1 Department of Neurology, Leeds General Infirmary, Leeds Teaching Hospitals NHS Trust, Leeds, UK; 2 Department of Electronics, University of York, York, UK; 3 Centre for Medieval Studies, University of York, York, UK; 4 Department of Neurosciences, Monash Medical Centre, Clayton, Australia; 5 Department of Medicine, Monash University, Clayton, Australia

**Keywords:** tremor, parkinson-s disease, dystonia, handwriting, archimedes spiral

## Abstract

When a patient presents with tremor, it can be useful to perform a few simple pen and paper tests. In this article, we explain how to maximise the value of handwriting and of drawing Archimedes spirals and straight lines as clinical assessments. These tasks take a matter of seconds to complete but provide a wealth of information that supplements the standard physical examination. They aid the diagnosis of a tremor disorder and can contribute to its longitudinal monitoring. Watching the patient’s upper limb while they write and draw may reveal abnormalities such as bradykinesia, dystonic posturing and distractibility. The finished script and drawings can then be evaluated for frequency, amplitude, direction and symmetry of oscillatory pen movements and for overall scale of penmanship. Essential, dystonic, functional and parkinsonian tremor each has a characteristic pattern of abnormality on these pen and paper tests.

## Introduction

In his Essay on the Shaking Palsy, James Parkinson referred to deterioration of handwriting.^1^ By the end of the 19th century, writing analysis had been developed as a clinical tool in neurology. David Marsden cited the example of handwriting to support his hypothesis that the basal ganglia are responsible for the implementation of learnt motor plans. Holding a felt-tipped pen in different ways, he showed how the character of his penmanship was conserved irrespective of the size of the script or the muscles used to execute it. He argued that the deeply engrained, highly reproducible motor planning of writing was particularly vulnerable to breakdown in Parkinson’s disease and other extrapyramidal disorders.^2^

Nearly all literate adults can write fluidly, having ‘over learnt’ handwriting in childhood and come to perform it automatically. Handwriting tasks, familiar and straightforward as they are, prove to be very useful diagnostic tools in tremor disorders. Tremor has its core characteristics of frequency, amplitude, direction and task specificity and may be conjoined with features such as bradykinesia, dystonia, ataxia and distractibility. The clinician’s challenge is to integrate all of this simultaneously occurring visual information during examination in order to classify a tremor. Simple pen and paper tests take a matter of seconds to complete. Having observed a patient during the *process* of writing and drawing, the form and content of the samples can be evaluated afterwards. These records of complex dynamic motor activity supplement the neurological examination and can track the progression of a tremor disorder or its response to therapy.

This paper explains how to maximise the usefulness of writing and drawing tasks in a busy clinic or by the bedside. Digitising tablets and other electronic tools can evaluate the pen movements in more detail but are beyond the scope of this article.

## Handwriting

**Figure 1:**
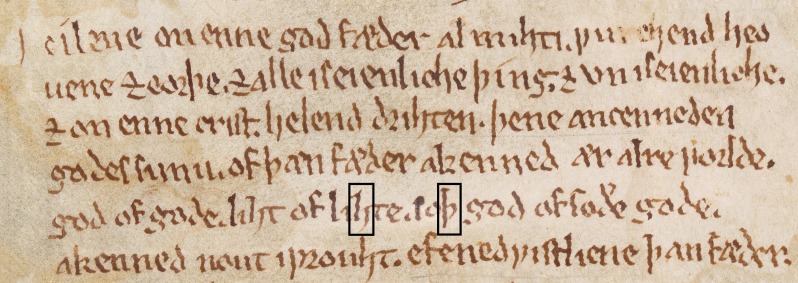
Medieval scripts. The handwriting of a 13th-century scribe known as ‘The Tremulous Hand of Worcester’ shows features consistent with essential tremor.^3^ There is a fine amplitude tremor of regular frequency, no micrographia, and the letters with long vertical sections such as ‘h’ and ‘þ’ (black boxes) demonstrate an 8–2 o’clock unidirectional tremor axis. (Detail of MS. Junius 121, fol. vi recto. The Bodleian Libraries, University of Oxford).

Cuneiform, the earliest known writing system, began before 3000 BC as pictograms impressed into clay with a pointed tool. Alphabetic writing arose in the Near East, developed by the Phoenicians and then adopted around the Mediterranean to form the basis of modern phonemic scripts. Until the invention of printing presses in the 15th century, handwriting was the only way of committing verbal information from memory to record. The natural histories of various tremors have been documented in medieval scripts (figure 1).^3 4^

Charcot examined the scripts of patients suffering from *paralysis agitans* with a magnifying glass, and he carefully studied their writing posture. He noted how tremulousness was chiefly expressed in the upstrokes of letters.^5^ Close associations between forensic writing analysis and neuroscience date from this period. Arthur Conan Doyle’s tales of Sherlock Holmes, which contain curious literary parallels with Charcot’s case histories, prefigure scientific methods in police detection that include questioned document analysis. Holmes’ assertion to Dr Watson in *The Adventure of the Reigate Squire* that a man’s handwriting could normally place him in the true decade of his lifespan is an exaggeration, however.^6^

## Archimedes spiral

**Figure 2:**
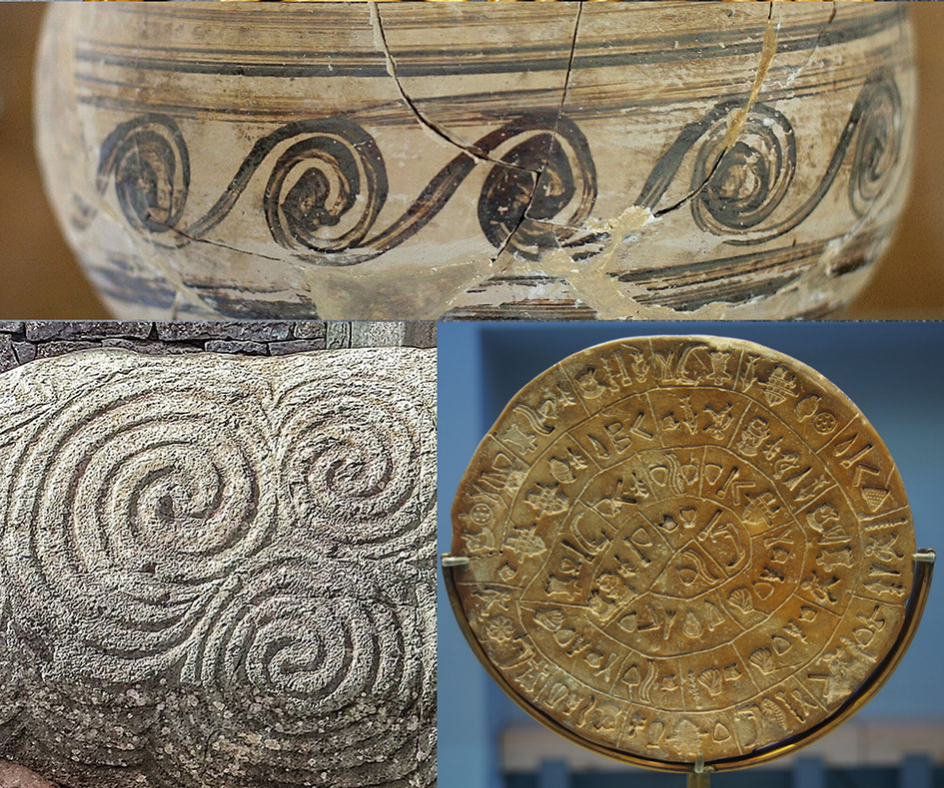
Decorative Archimedes spirals. Top: detail of spirals on a late Bronze Age clay jug from Akrotiri, Santorini (Wikimedia Commons, user: Zde). Bottom left: triple spirals on the entrance stone of the prehistoric monument at Newgrange, Ireland (Wikimedia Commons, user: Jal74). Bottom right: the winding spiral of the Phaistos Disc, a clay artefact dating from the second millennium BC (Wikimedia Commons, user: C messier).

This spiral is named after the Greek polymath Archimedes (287–212 BC), having appeared in his 225 BC essay *On Spirals*. The shape had actually been described a few years earlier by his friend Conon of Samos (280–220 BC), a Greek astronomer who named the star constellation *Coma Berenices*. The spiral’s formula r = a+bθ gives a constant separation distance between each turn. The Archimedes spiral has been a decorative motif since prehistory, appearing on pottery and other artefacts from the Neolithic and Bronze Ages (figure 2). Spiral shapes seen in nature (mollusc shells, plants, cyclonic weather systems, galaxies) are different, usually having logarithmic forms.

The Archimedes spiral drawing captures the frequency, amplitude and direction of a tremor without the necessity of allowing for stylistic differences of handwriting. Because it demands one continuous pen movement without the brief breaks between writing words, the spiral emphasises the abnormal movements of dystonia, hypokinesia and tremor.

## Vertical and horizontal line drawings

This task complements the other two as nearly all adults can hold the pen well enough with each hand for valid side-to-side comparisons. The longer, straighter pen strokes enable an estimate of tremor frequency to be made and are quite sensitive to tremor variability, as is often present in a functional tremor.

## How to do it

**Figure 3:**
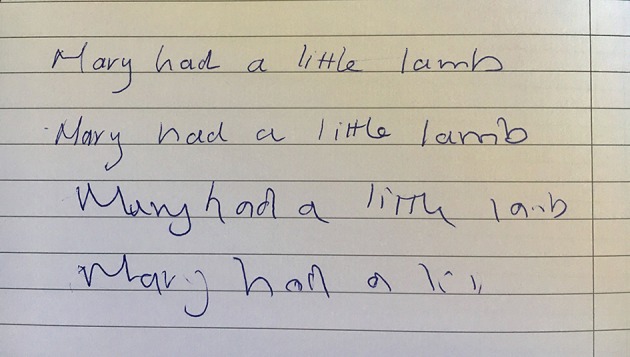
Longer handwriting samples may be required. This patient had mild writer’s cramp that was only apparent with quick repetition of the writing task. The first two lines of writing were executed reasonably normally but there was progressive flexor posturing of the wrist and an obvious deterioration in the quality of the letter formations by the third and fourth lines.

There is no right or wrong way, but we begin by asking the patient to write *Mary had a little lamb* with the dominant hand. Performing this once is often enough, but there are circumstances in which repetition will pay dividends. Dystonic posturing of the wrist and fingers may become more obvious with longer periods of writing (figure 3). Decrementing micrographia in Parkinson’s disease occasionally needs an extended writing task to manifest. Thereafter, we draw an Archimedes spiral and ask the patient to copy it, first with the dominant hand and then the non-dominant. Some clinicians prefer to demonstrate the task by tracing a spiral in the air, leaving the patient to determine the size of the drawing. Lastly, we instruct the patient to draw horizontal and vertical lines with each hand; the lines should be at least 10 cm long. Repetition of the drawing tasks may also be useful—in suspected functional tremor to look for variability or in cases of subtle or episodic tremor to increase the chance of recording it. If nothing is visible, it may help to ask the patient to lift their hand off the paper while performing each task. This tends to amplify a tremor and any associated posturing. Each tremor condition has a distinctive pattern with writing and drawing. Often these aberrations are seen across all the pen and paper tasks but sometimes they will only be visible in one. Variability between the tasks may itself be diagnostically useful. It is best to perform all three.

## The writing and drawing tasks in specific tremor disorders

### Essential tremor

One of the hardest differentiations to make, even for experienced movement disorder specialists, is between early essential tremor and mild bilateral dystonic tremor. Pen and paper tasks can be particularly useful here. Analysis of spirals drawn by people with essential tremor found that the majority demonstrate a unidirectional tremor axis independent of which part of the spiral is being drawn.^7 8^ This reflects the distal flexion–extension tremor movements, predominantly involving the wrists and fingers. The axis of essential tremor is usually in the 8–2 o’clock direction for right hand spiral drawings and 10–4 o’clock for the left hand.^8^ The unidirectional axis is also seen in handwriting, especially in letters that have vertical linear strokes such as ‘l’, ‘h’ and ‘p’. In contrast to Parkinson’s disease, the size of spirals and handwriting tend to be normal; see table 1.

**Table 1:** Typical characteristics of tremor types seen in writing and drawing tasks

	Essential tremor	Dystonic tremor	Parkinson’s disease	Functional tremor
Handwriting				
Size	Normal or large	Normal	Usually small	Variable
Tremor features	Regular amplitude and frequency	Irregular jerky amplitude and frequency	Regular amplitude and frequency	Variable; often no tremor in writing
Tremor intrusion in letter sections	Vertical letter strokes; unidirectional axis	All sections of letters; multidirectional axis	Vertical letter strokes; unidirectional axis	Variable
Progressive deterioration	No	Yes—shape of letters worsens due to posturing	Sometimes—size of letters may decrement	Not usually
Pen pressure	Normal	Hard pressure	Normal	Normal
Spirals				
Size	Normal	Normal	Small	Variable
Spacing of turns	Normal (maybe wider)	Normal (maybe tighter)	Tighter	Variable
Tremor axis	Unidirectional	Multidirectional	Unidirectional	Variable
Tremor frequency	Regular	Irregular/jerky	Regular	Variable
Progressive deterioration	No	Sometimes—more tremor and pressure	No	Variable tremor frequency, axis and amplitude
Straight lines				
Tremor axis	Unidirectional Right: 8–2 o’clock Left: 10–4 o’clock	Multidirectional	Unidirectional Right: 8–2 o’clock Left: 10–4 o’clock	Variable
Tremor frequency	Regular	Irregular	Usually regular	Variable
Tremor amplitude	Small, regular	Small, irregular	Small, regular	Large, irregular
Symmetry	Symmetrical	Asymmetrical	Asymmetrical	Variable
Other features		Writing may be worse than drawing tasks	Longer duration to compete task (bradykinesia)	Straight lines usually most impaired task

**Figure 4:**
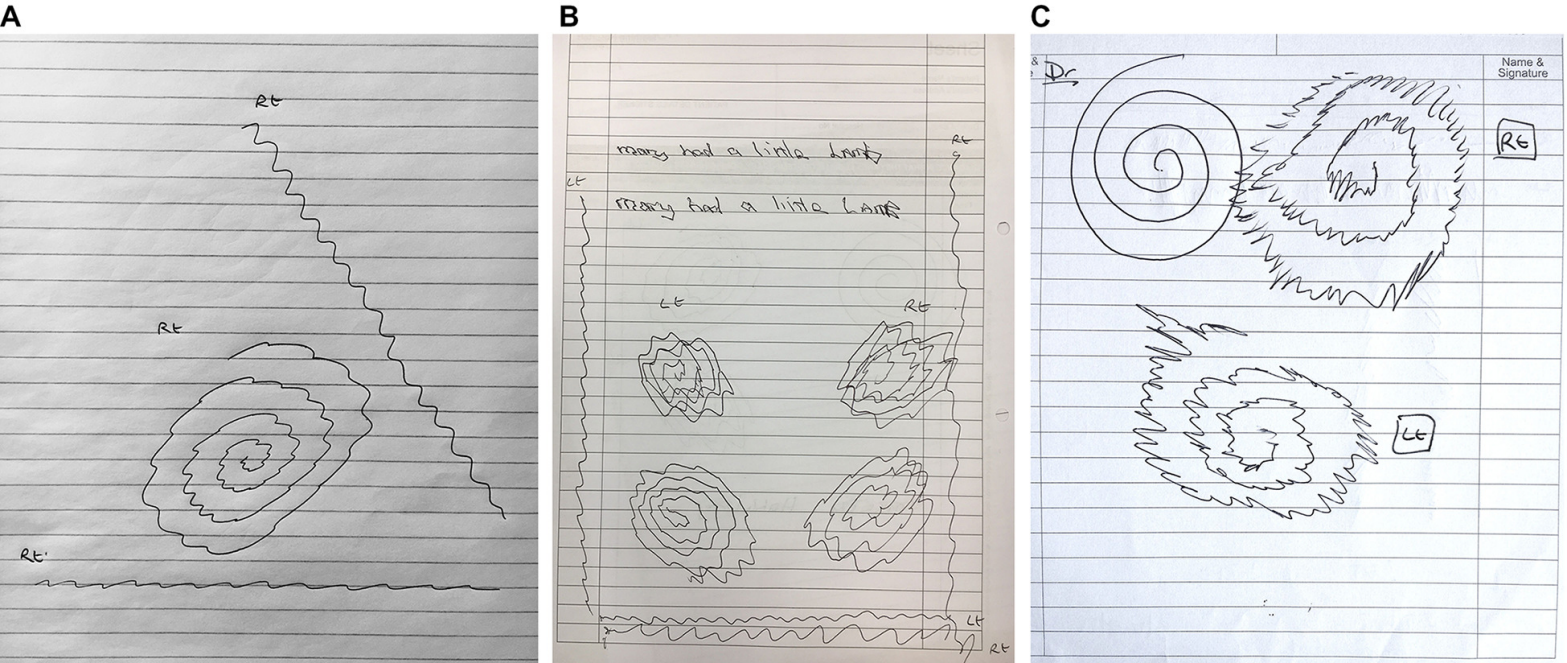
Essential tremor. (A) The Archimedes spiral drawing shows a unidirectional tremor axis in the 8–2 o’clock direction, suggesting essential tremor, but it is not clear whether the amplitude and frequency are constant. Both straight lines show the frequency to be regular; the line drawn perpendicular to the tremor axis emphasises the amplitude and makes it easier to discern that it is also constant. (B) The handwriting is tremulous and potentially compatible with either essential tremor or dystonic tremor. The spirals show a unidirectional 8–2 o’clock axis in the right hand spirals and a 10–4 o’clock axis in the left hand, symmetrical in size and severity—all features that point towards essential tremor. However, the regularity of the amplitude and frequency is difficult to determine from the spirals as the severity of the tremor causes the turns to overlap. The straight line drawings demonstrate that the amplitude, frequency and axis are all constant. The left (dominant) handed vertical line has 18 oscillations drawn over 2 s, giving an estimated frequency of 9 Hz. (C) Spiral drawings from a patient with severe essential tremor showing large tremor oscillations with a unidirectional axis, fairly regular amplitude and frequency, and symmetry between the hands. An enlarged spiral is sometimes seen in severe essential tremor, perhaps reflecting ataxia that may occur as part of the disorder, or to compensate for the size of the penstroke undulations.

At the point of diagnosis, essential tremor oscillations typically have a small amplitude (<1 cm) and a regular high frequency (8–12 Hz), with each hand equally affected (figure 4). One method to clarify tremor variability is to ask the patient to draw a line at a steady rate and in a direction perpendicular to the tremor axis of the spiral (figure 4a). A longer tracing of the tremor makes it easier to see whether the amplitude and frequency are constant (usual in essential tremor) or variable (more suggestive of dystonic or functional tremor). It is possible to calculate the tremor frequency by asking the patient to draw a straight line for about 2 s timed with the digital stopwatch of a mobile phone. The number of complete oscillations of the line divided by the exact interval between ‘start’ and ‘stop’ commands from the digital display gives the approximate frequency in hertz (figure 4b).

### Dystonic tremor

In contrast to essential tremor, there is often a multidirectional axis in dystonic tremor. Archimedes spiral drawings are particularly good at highlighting this feature though it is usually apparent in writing and line drawing too. The direction of the oscillations varies because dystonic tremor involves co-contraction of agonist and antagonist muscles affecting proximal (shoulder/elbow) as well as distal (wrist/digits) parts. The commonly observed exacerbation of dystonic tremor with certain postures and an overflow of muscle activity with voluntary actions are other contributing factors. It should be noted that tremor axis analysis from Archimedes spirals does not conclusively discern essential from dystonic tremor. A study of 135 individuals with familial essential tremor found that a unidirectional axis in two or more of four copied spirals (two from each hand) classified 67.5% of essential tremor cases and 60% of those with dystonic tremor.^7^

**Figure 5:**
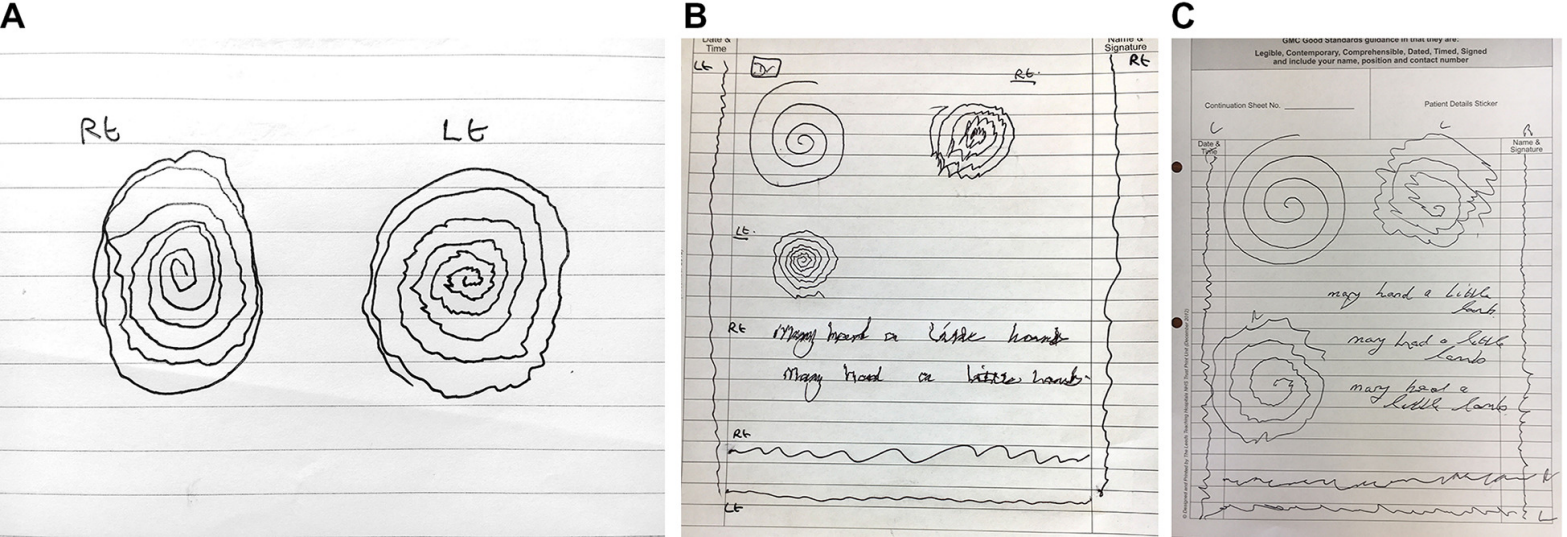
Dystonic tremor. (A) Oscillations occur in all sections of the spirals, denoting a multidirectional axis. The amplitude and frequency also vary, giving a jerky appearance. (B) There is evidence of forceful pen pressure consistent with dystonic posturing in the spiral drawings and handwriting samples. We have observed that many patients with dystonia have a tendency to draw more than three turns of the spiral. The left-handed spiral demonstrates a multidirectional tremor axis. Straight lines drawn at a steady rate, particularly the vertical one, show that the frequency is variable. On horizontal line and spiral drawings, the tremor amplitude is asymmetric, being larger on the right. (C) The spiral and line drawings demonstrate a multidirectional tremor axis with a jerky pattern caused by variable amplitude and frequency—all features consistent with dystonic tremor. In this case, the handwriting is normal with very little tremor intrusion.

**Figure 6:**
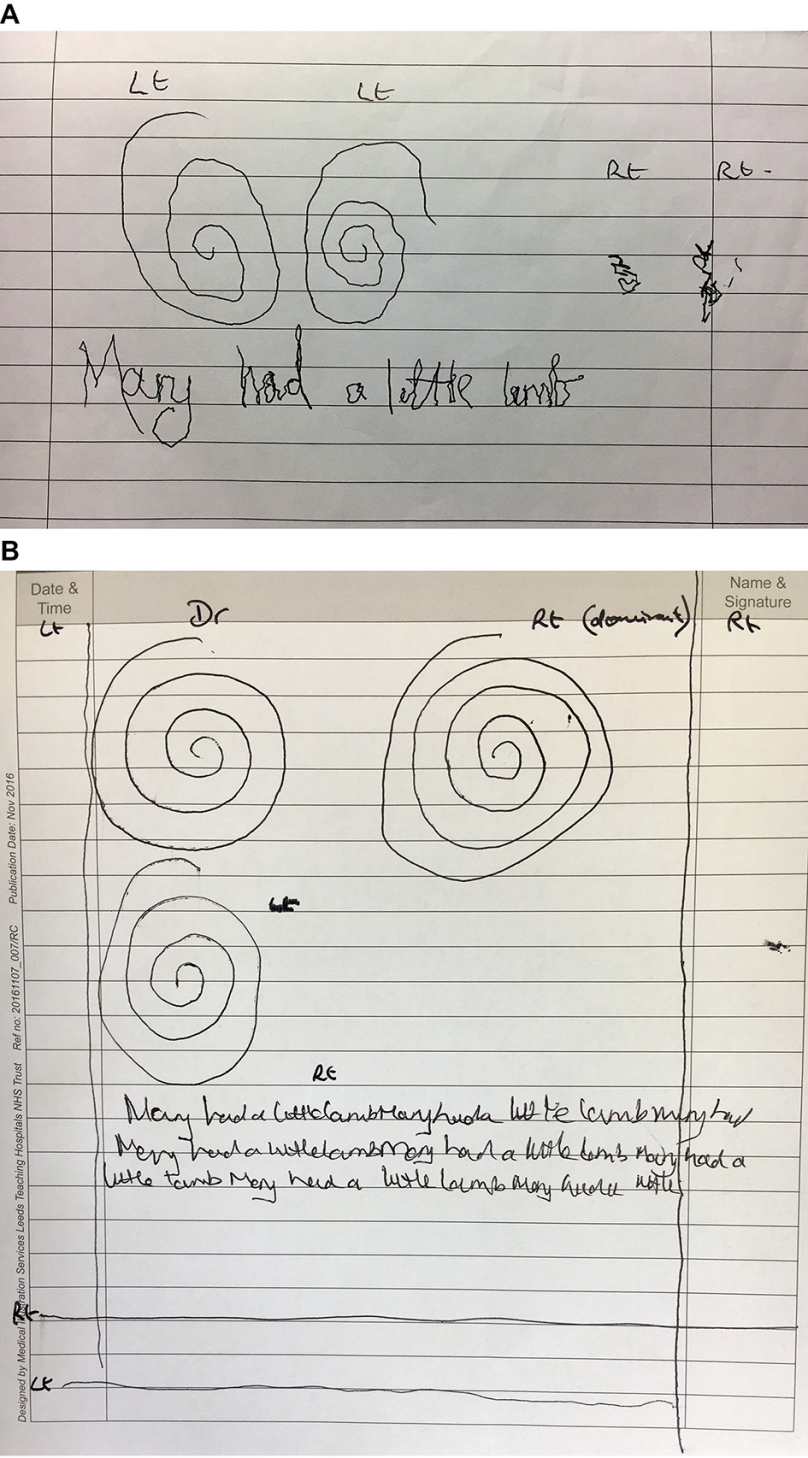
Dystonic tremor with writer’s cramp. (A) Severe: the patient is barely able to hold a pen with the right (dominant) hand because of involuntary hand posturing. He had been using non-dominant left hand to write but there is now evidence of dystonia—writer’s cramp and dystonic tremor—on this side too. The spiral drawings and handwriting with the left hand have a multidirectional axis with evidence of lighter ink pressure because there is dystonic extensor posturing of the fingers and wrist. (B) Mild: the patient had minimal tremor and mild dystonia that only became apparent with prolonged periods of writing. Note how the writing progressively deteriorates on the second and third lines. Spiral and straight line drawings were normal.

Further clues may be obtained by looking at the tremor amplitude; in dystonic tremor, it fluctuates and this variability in both axis and amplitude produces a ‘jerky’ pattern. The tremor is usually asymmetric and of lower frequency (<7 Hz) than essential tremor. Abnormal posturing of the hand and arm may occur. This can result in unusually hard pressure being applied to the pen or even an inability to draw or write altogether, as is seen in severe writer’s cramp (figures 5 and 6). Sometimes patients with dystonic tremor find writing much more difficult than drawing, reflecting the task-specific nature of dystonia.

### Parkinson’s disease

People with Parkinson’s disease often write and draw slowly, producing small handwriting and spirals formed with tightly bunched turns. The reduced size and speed of pen movements reflects bradykinesia, the cardinal motor sign of Parkinson’s disease. The authors find spiral drawings to be more sensitive than subtle micrographia, especially if small handwriting is a lifelong trait. Decrementing bradykinesia—a gradual reduction in both speed and size of writing—occurs in some but not in all patients with Parkinson’s disease.^9^

**Figure 7:**
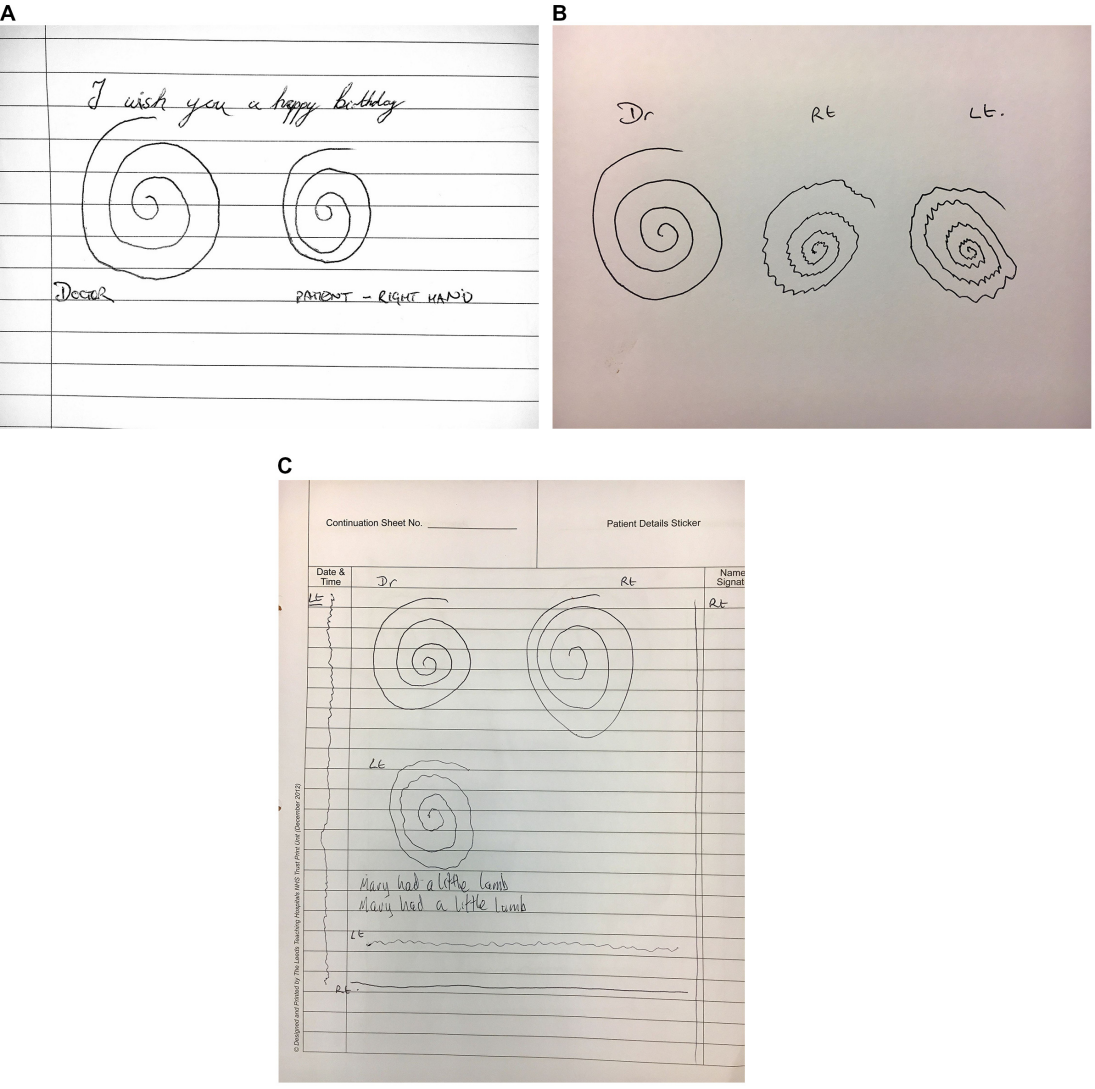
Parkinson’s disease. (A) The spiral of the patient with Parkinson’s disease (right) is smaller than the clinician’s, with tighter turns. There is no evidence of action tremor. (B) This patient presented with severe bilateral tremor that made it difficult to assess for bradykinesia and rigidity in the upper limbs. It was initially mistaken for essential tremor. There are tightly bunched turns with a unidirectional axis and the patient was also slow to copy the spiral. The oscillations become more widely spaced as the patient speeds up towards the outer sections of the spiral–a pattern frequently seen in Parkinson’s disease, probably reflecting the difficulty with movement initiation. (C) The writing and spiral with the right hand are within normal limits. The left-handed spiral shows a jerky fine amplitude tremor with slight reduction in overall size. The line drawings show a more jerky variation in amplitude and frequency than is usually seen in Parkinson’s disease. This patient has an atypical Parkinson’s action tremor.

Action tremor is present in nearly half of patients with Parkinson’s disease when drawing (figure 7).^10^ While the axis of a parkinsonian tremor is similar to that of essential tremor during Archimedes spiral drawing, the reduced diameter and increased density of turns differentiate tremulous Parkinson’s disease with reasonable specificity.^11^

### Functional tremor

**Figure 8:**
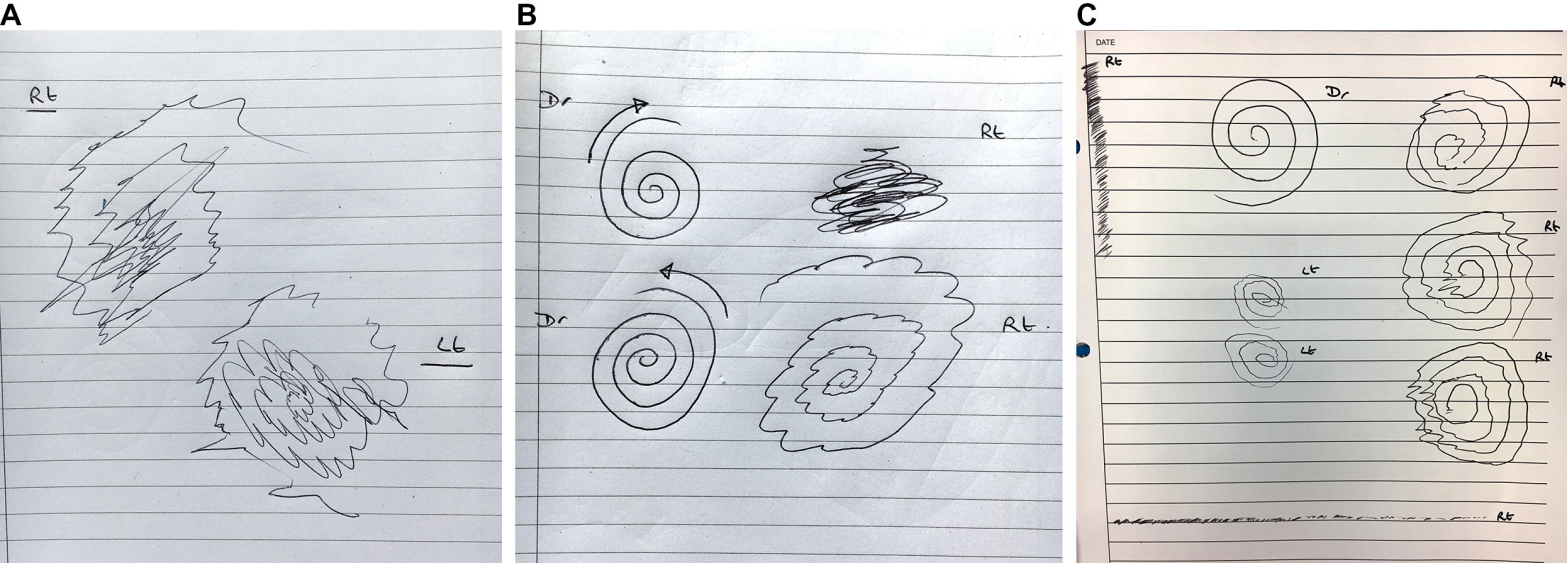
Functional tremor. (A) The tremor affects both hands but there is variation in amplitude and frequency between the right and left spirals. (B) There is a marked discrepancy when the patient copies the spiral in alternate directions. (C) This patient presented with a right (dominant) hand tremor that was consistently unidirectional (8–2 o’clock direction). Other features were variable and inconsistent, suggesting a functional disorder. The site of tremor intrusion changed within a single spiral and between consecutive spirals; the amplitude changed from small to large within a single spiral but there was a consistent amplitude during straight line drawing; while the spirals were drawn quickly, there was freezing of drawing for straight lines. Note the increased density of oscillations, especially on the vertical line drawing.

A key feature of functional tremor is inconsistency, so it is important to look for this sign across repeated tests of the same task, as well as comparing the tremor between different writing and drawing tasks. While dystonic tremor has some activity dependence, functional tremor shows the most intraspiral and interspiral variability.^12^ The tremor frequency may change *within* an individual spiral or between those executed by left and right hands; anticlockwise spiral copying may be impaired, whereas clockwise drawings are unaffected. The authors observe that functional tremor is usually more severe during line drawing than the other two tasks. Sometimes, there is no tremor at all with the spirals or writing but florid tremor is seen when line-copying (figure 8). Drawing tasks can be particularly useful when discussing the diagnosis of functional tremor by providing objective evidence of functional signs. It is easier to demonstrate this to a patient than dynamic findings such as tremor entrainment or distractibility.

## Longitudinal monitoring

**Figure 9:**
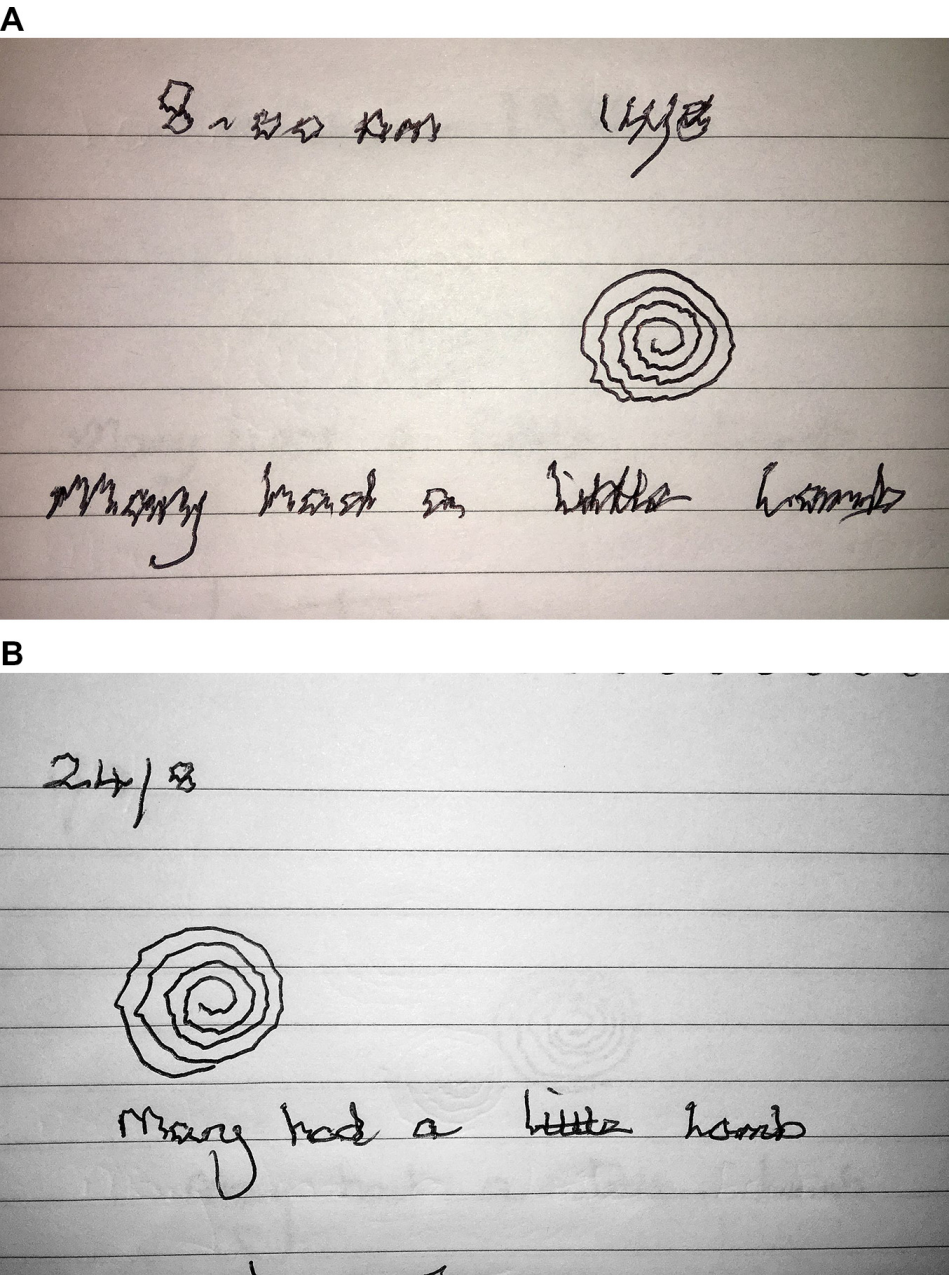
Monitoring response to treatment. (A) Before: the spirals and handwriting show features consistent with dystonic tremor—a multidirectional jerky tremor, hard pen pressure and progressive deterioration in handwriting legibility due to flexor posturing. (B) After: the same tasks recorded after botulinum toxin injections were administered to the forearm flexor muscles documents a clear improvement.

Serial recording of handwriting, Archimedes spirals and line drawings monitor how a tremor changes over time. There may be examples of handwriting from years past (diaries, Christmas cards, letters) that record its early history. These pen and paper tasks can especially help when comparing responses to sequential drug trials; for instance, whether propranolol has been more effective at reducing an essential tremor than primidone (figure 9). Visual evaluation of the spirals can be quantified by applying Bain and Findley’s spirography scale, which provides a 10-point numeric measure of the severity of tremor.^13^ This has been shown to correlate well with digitising tablet analysis, have good interrater and intrarater reliability, and respond to change over time.^14–16^ When interpreting longitudinal records collected over many years, it is important to remember that the natural history of most tremor disorders is for the amplitude to gradually increase with a reduction in frequency.

**Key points**

- These three pen and paper tasks—handwriting, Archimedes spirals and line drawings—are quick to perform, provide objective evidence of abnormal neurological signs and can help in the differential diagnosis of tremor.
- To differentiate essential tremor from dystonic tremor, look for these features: essential tremor is usually a higher frequency, smaller amplitude, symmetrical tremor with a *single* axis; dystonic tremor has a lower frequency, a more variable or jerky amplitude, more asymmetry, a *multidirectional* axis and more forceful pen pressure.
- The writing and drawing of patients with Parkinson’s disease tends to be smaller and slower, with tightly bunched letters and curves; it often exhibits a unidirectional asymmetric tremor.
- Marked intertask and intratask variability when performing the pen and paper assessments suggests functional tremor.
- Handwriting, Archimedes spirals and line drawing are useful methods to monitor the progression of a tremor disorder or for assessing its response to treatment.
